# Ceramic-reinforced HEA matrix composites exhibiting an excellent combination of mechanical properties

**DOI:** 10.1038/s41598-022-25734-w

**Published:** 2022-12-12

**Authors:** M. Adil Mehmood, Khurram Shehzad, M. Mujahid, Talha Bin Yaqub, Andy Godfrey, Filipe Fernandes, F. Z. Muhammad, Khurram Yaqoob

**Affiliations:** 1 School of Chemical and Materials Engineering (SCME), National University of Sciences and Technology (NUST), H-12, Islamabad, Pakistan; 2 Department of Mechanical Engineering, CEMMPRE - Centre for Mechanical Engineering Materials and Processes, University of Coimbra, Rua Luís Reis Santos, 3030-788 Coimbra, Portugal; 3 School of Materials Science and Engineering, Tsinghua University, Beijing, China; 4 ISEP - School of Engineering, Polytechnic of Porto, Rua Dr. António Bernardino de Almeida 431, 4200-072 Porto, Portugal; 5 CESAT, Islamabad, Pakistan

**Keywords:** Engineering, Materials science

## Abstract

CoCrFeNi is a well-studied face centered cubic (fcc) high entropy alloy (HEA) that exhibits excellent ductility but only limited strength. The present study focusses on improving the strength-ductility balance of this HEA by addition of varying amounts of SiC using an arc melting route. Chromium present in the base HEA is found to result in decomposition of SiC during melting. Consequently, interaction of free carbon with chromium results in the in-situ formation of chromium carbide, while free silicon remains in solution in the base HEA and/or interacts with the constituent elements of the base HEA to form silicides. The changes in microstructural phases with increasing amount of SiC are found to follow the sequence: fcc → fcc + eutectic → fcc + chromium carbide platelets → fcc + chromium carbide platelets + silicides → fcc + chromium carbide platelets + silicides + graphite globules/flakes. In comparison to both conventional and high entropy alloys, the resulting composites were found to exhibit a very wide range of mechanical properties (yield strength from 277 MPa with more than 60% elongation to 2522 MPa with 6% elongation). Some of the developed high entropy composites showed an outstanding combination of mechanical properties (yield strength 1200 MPa with 37% elongation) and occupied previously unattainable regions in a yield strength versus elongation map. In addition to their significant elongation, the hardness and yield strength of the HEA composites are found to lie in the same range as those of bulk metallic glasses. It is therefore believed that development of high entropy composites can help in obtaining outstanding combinations of mechanical properties for advanced structural applications.

## Introduction

High entropy alloy design is a promising new concept in the field of metallurgy^1,2^. High entropy alloys (HEAs) have been shown in some cases to exhibit an outstanding combination of physical and mechanical properties, including high thermal stability^3,4^, superplastic elongation^5,6^, fatigue resistance^7,8^, corrosion resistance^9–11^, excellent wear^12–15^ and tribological properties^15–17^ and good mechanical performance even at high temperatures^18–22^ and cryogenic temperatures^23–25^. Outstanding combinations of mechanical properties in HEAs are generally attributed to the presence of four core effects, namely high configurational entropy^26^, severe lattice distortion^27^, sluggish diffusion^28^ and cocktail effects^29^. HEAs are generally characterized as being of FCC, BCC, and HCP type. FCC HEAs typically contain transition elements such as Co, Cr, Fe, Ni and Mn, and exhibit excellent ductility (even at cryogenic conditions^25^) but have low strength. BCC HEAs generally consist of high-density elements, such as W, Mo, Nb, Ta, Ti and V exhibit very high strength, but have low ductility and low specific strength^30^.

Microstructural modifications of HEAs based on mechanical processing, thermomechanical processing and elemental additions have been explored in order to obtain better combinations of mechanical properties. Severe plastic deformation of the CoCrFeMnNi FCC HEA through high pressure torsion was found to result in a large increase in both hardness (520 HV) and strength (1950 MPa), but the development of nano-crystalline microstructure (~ 50 nm) made the alloy brittle^31^. Introduction of twinning-induced plasticity (TWIP) and transformation-induced plasticity (TRIP) to the CoCrFeMnNi HEA was found to impart good strain-hardening ability, resulting in a large tensile ductility, albeit at the cost of low values of true ultimate tensile strength (1124 MPa)^32^. The use of shot peening to develop a hierarchical microstructure (consisting of a thin deformed layer and an un-deformed core) in a CoCrFeMnNi HEA resulted in a strength increase, but the improvement was limited to only around 700 MPa^33^. The development of multi-phase high entropy alloys and eutectic high entropy alloys using non-equiatomic elemental additions has also been explored in the quest for materials with better combinations of strength and ductility^34–41^. A fine distribution of hard and soft phases in eutectic high entropy alloys was indeed found to result in relatively better combinations of strength and ductility^35,38,42,43^.

CoCrFeNi system is extensively studied single-phase fcc high entropy alloy. This system has rapid strain hardening characteristic^44^ and excellent ductility both at cryogenic and elevated temperatures^45,46^. Different attempts have been made to improve its relatively lower strength (~ 300 MPa)^47,48^, which include grain refinement^25^, multi-phase microstructure^49^, precipitation^50–52^, and transformation induced plasticity (TRIP)^53^. Grain refinement of the as-cast fcc CoCrFeNi HEA by heavy cold-drawing resulted in an increased strength from ~ 300 MPa^47,48^ to 1.2 GPa^25^, but with a loss of ductility from more than 60% to 12.6%. Multi-phase microstructure developed in CoCrFeNi HEA by the addition Al increased its yield strength to 786 MPa with an elongation of around 22%^49^. Precipitation strengthening due to formation of precipitates with Ti and Al addition in the CoCrFeNi HEA increased its yield strength to 645 MPa with an elongation of 39%^51^. TRIP mechanism (fcc → hcp martensitic transformation) and twinning increased tensile strength of CoCrFeNi HEA to 841 MPa with 76% fracture elongation^53^.

Addition of ceramic reinforcements to the fcc matrix HEA have also been tried for the development of high entropy composites that can exhibit better combination of strength and ductility. High entropy composites have been developed by vacuum arc melting^44^, mechanical alloying^45–48,52,53^, spark plasma sintering^46,51,52^, vacuum hot pressing^45^, hot iso-static pressing^47,48^ and additive manufacturing^43,50^ processes. Carbides, oxides and nitrides such as WC^44–46^, Al_2_O_3_^47^, SiC^48^, TiC^43,49^, TiN^50^ and Y_2_O_3_^51^ have been used as ceramic reinforcement for the development of HEA composites. Selection of a suitable HEA matrix and ceramic phase is particularly important to design and develop strong and tough HEA composites. In the present work CoCrFeNi has been selected as the matrix material. Varying amounts of SiC have been added to CoCrFeNi HEA and their effect on the microstructure, phase structure and mechanical properties have been investigated.

## Materials and methods

### Design and preparation of alloys

High purity Co, Cr, Fe and Ni metals (99.95 wt.%) in the form of elemental pellets and SiC powder (purity 99%, size -400 mesh) were used as raw materials for the development of the HEA composites. An equiatomic CoCrFeNi HEA composition was first placed in a hemispherical water-cooled Cu mold, after which the chamber was evacuated to 3 × 10^–5^ mbar. High purity argon was introduced to obtain the desired vacuum level for arc melting using a non-consumable W electrode. The as-produced ingot buttons were flipped over and re-melted five times to ensure good homogeneity. High entropy composites of different composition were prepared by adding amounts of SiC to as produced equiatomic CoCrFeNi buttons, in each case re-homogenized by flipping over and re-melting five times. The as-cast buttons of the resulting composites were sectioned using electrical discharge machining for further testing and characterization. Samples for microstructural studies were prepared using standard metallographic procedures. The samples were examined initially using an optical microscope (Leica Microscope DM6M), where the Leica image analysis software (LAS Phase Expert) was used for quantitative phase analysis. Three images taken in different regions were selected for the phase analysis, covering a total area of around 27,000 µm^2^. Further detailed microstructural studies, including chemical composition analysis and elemental distribution analysis, were carried in a scanning electron microscope (JEOL JSM-6490LA) equipped with an energy dispersive spectroscopy (EDS) analysis system. Crystal structure characterization of the HEA composites was carried out using an X-ray diffraction system (Bruker D2 phaser), operated with a CuKα source at a step size of 0.04°. Vickers micro-hardness tests and compression tests were carried out to study the effect of microstructural changes on the mechanical properties of the HEA composites. For the hardness tests a load of 500 N was applied for 15 s, using a minimum of 10 indents for each sample. Room temperature compression testing of the HEA composites was carried on rectangular shaped specimens (7 mm × 3 mm × 3 mm) using a SHIMADZU 50KN universal testing machine (UTM) at an initial strain rate of 0.001/s.

## Results and discussion

**Figure 1:**
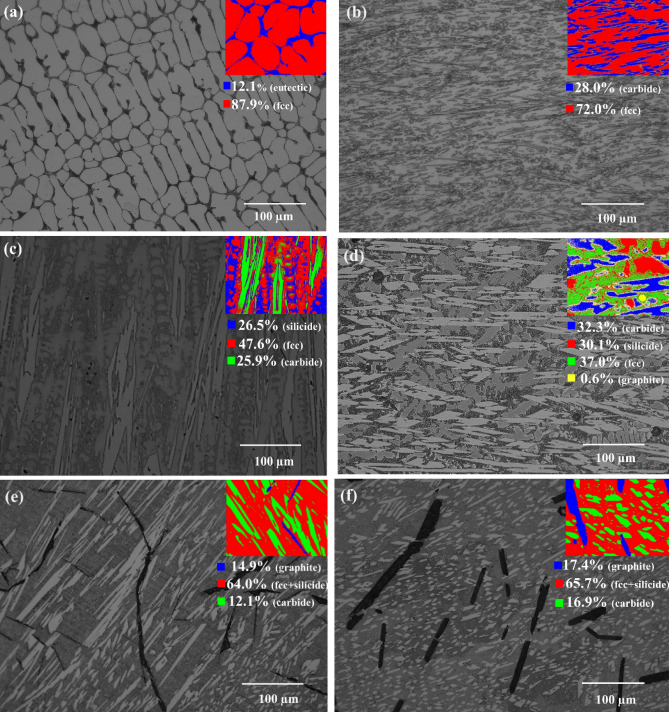
Optical micrographs of the as-developed high entropy composites: (**a**) S-1, (**b**) S-2, (c) S-3, (**d**) S-4, (**e**) S-5 and (**f**) S-6. The inset images show an example phase analysis results based on image contrast using the LAS Phase Expert software.

High entropy composites were prepared by addition of 3%, 6%, 9%, 12%, 15% and 17% SiC (all wt. %) to the CoCrFeNi matrix and are referred to hereafter as samples S-1 to S-6, respectively. The reference sample without SiC addition is referred to hereafter as sample S-0. Optical micrographs of the as-developed HEA composites are shown in Fig. 1, where the single-phase microstructure of the CoCrFeNi HEA changes to a microstructure consisting of multiple phases with different morphology, size and distribution, as a result of the addition of different amounts of SiC to the composition. The amount of each phase was determined based on image analysis using the LAS Phase Expert software. An example region of this analysis is shown in the inset images (upper right corner) in Fig. 1, together with the area fraction of each phase component.

As shown in Fig. 1a, a eutectic microstructure develops between matrix volumes in the S-1 composite where the amounts of matrix and eutectic phase were estimated to be 87.9 ± 0.47% and 12.1% ± 0.51%, respectively. In the composite (S-2), shown in Fig. 1b, there was no longer any evidence of a eutectic reaction during solidification and a completely different microstructure from the S-1 composite was observed. The microstructure of the S-2 composite was relatively refined, consisting of fine plates (carbides), uniformly distributed in the matrix phase (fcc). The volume fractions of matrix and carbides were estimated to be 72 ± 1.69% and 28 ± 1.69%, respectively. A new phase (silicide), in addition to the matrix and carbides, was found in the S-3 composite, as shown in Fig. 1c, where the volume fractions of this silicide, carbide and the matrix phase were estimated to be about 26.5 ± 0.41%, 25.9 ± 0.53 and 47.6 ± 0.34, respectively. Yet another new phase (graphite) was observed in the microstructure of the S-4 composite, where a total of four phases were identified. The graphite phase has a well-defined globular form, with dark contrast in optical images, and was present only in a small amount (estimated as a volume fraction of only about 0.6 ± 0.30%). In the S-5 and S-6 composites, only three phases were identified, with the dark-contrast graphite phase appearing in these composites in flake-like form. The graphite flakes in the S-6 composite were wider and shorter compared to the graphite flakes in the S-5 composite, with more regular appearance. A corresponding increase in the amount of the graphite was also observed, from 14.9 ± 0.85% in the S-5 composite to around 17.4 ± 0.55% in the S-6 composite.

**Figure 2:**
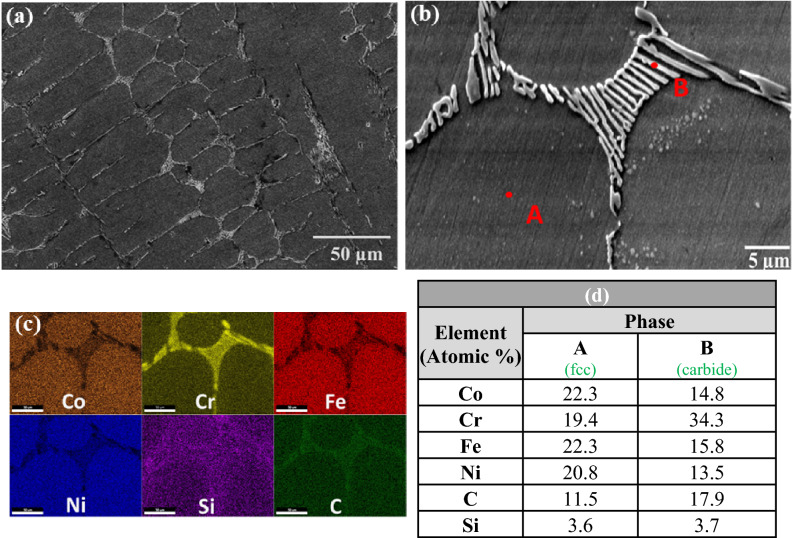
(**a**) SEM image of sample S-1, (**b**) magnified view, (**c**) elemental mapping, (**d**) EDS results at the indicated positions.

To further investigate the detailed microstructure and chemical content of the phases present in the HEA composites the samples were examined in the SEM, where EDS point analysis and chemical mapping were carried out. Results for the S-1 composite are shown in Fig. 2, where the presence of a eutectic mixture separating regions of the majority matrix phase is clearly seen. Chemical mapping of the S-1 composites, shown in Fig. 2c, indicated a uniform distribution of Co, Fe, Ni, and Si in the matrix phase. However, a smaller amount of chromium was found in the matrix phase in comparison with the other elements of the base HEA, indicating diffusion of Cr out of the matrix. The component of the eutectic phase appearing white in the SEM images was found to be rich in chromium and carbon, suggest that this is a carbide of chromium. The absence of discrete SiC particles in the microstructure, combined with the observation of a lower amount of chromium in the matrix, and with presence of eutectic mixture containing a chromium rich phase indicates complete decomposition of SiC during melting. As a result of the SiC decomposition, silicon was found to be dissolved in the matrix phase, while free carbon interacted with chromium to form chromium carbide. It can be noted that through EDS only the qualitative determination of carbon was only taken, and the phase formation were confirmed through identification of characteristic carbide peaks in the XRD patterns.

**Figure 3:**
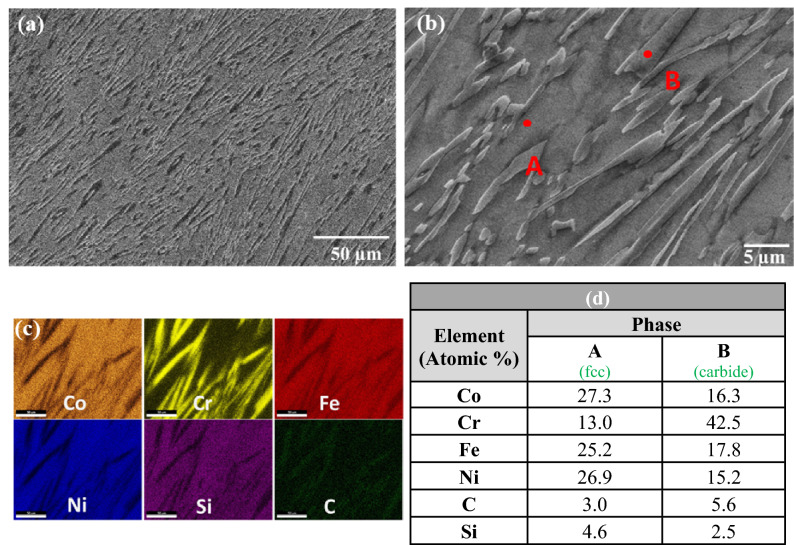
(**a**) SEM image of sample S-2, (**b**) magnified view, (**c**) elemental mapping, (**d**) EDS results at the indicated positions.

Analysis of the S-2 composite is shown in Fig. 3. Similar to the appearance in the optical microscope, SEM inspection revealed a fine-scale structure consisting of just two phases, with the presence of a fine plate like phase uniformly distributed throughout a matrix phase, and an absence of the eutectic phase. Elemental mapping and EDS point analysis of the plate-like phase indicated the presence of relatively high amounts of Cr (yellow) and C (green) in this phase, again indicating decomposition of SiC during melting and interaction of the released carbon with chromium in the HEA matrix to form a plate-type carbide phase. Elemental mapping and point analysis of the matrix phase revealed that most of the cobalt, iron, nickel and silicon were present in the matrix phase.

**Figure 4:**
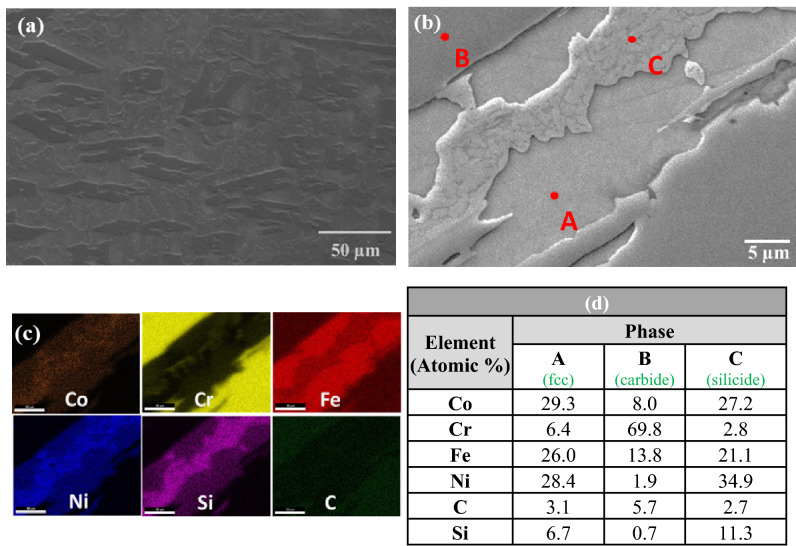
(**a**) SEM image of sample S-3, (**b**) magnified view, (**c**) elemental mapping, and (**d**) EDS results at the indicated positions.

SEM investigation of the S-3 composite revealed the presence of a new phase in addition to the carbide phase and matrix phases. Elemental mapping, Fig. 4c, and EDS point analysis, Fig. 4d, indicated that this new phase was rich in nickel, cobalt and silicon.

**Figure 5:**
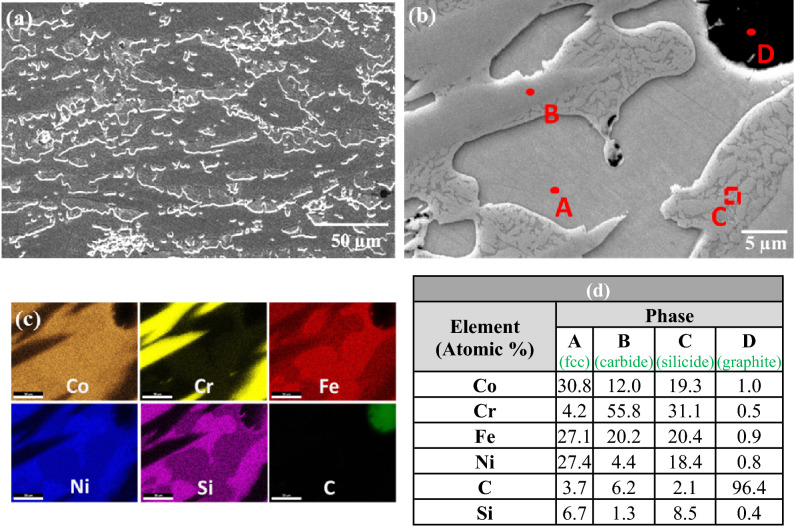
(**a**) SEM image of sample S-4, (**b**) magnified view, (**c**) elemental mapping, and (**d**) EDS results at the indicated positions.

The results of SEM and EDS analysis of the S-4 composites are shown in Fig. 5. In addition to the three phases seen in the S-3 composite, the presence of graphite globules was also found. The volume fraction of the silicon-rich phase was also higher than in the S-3 composite.

**Figure 6:**
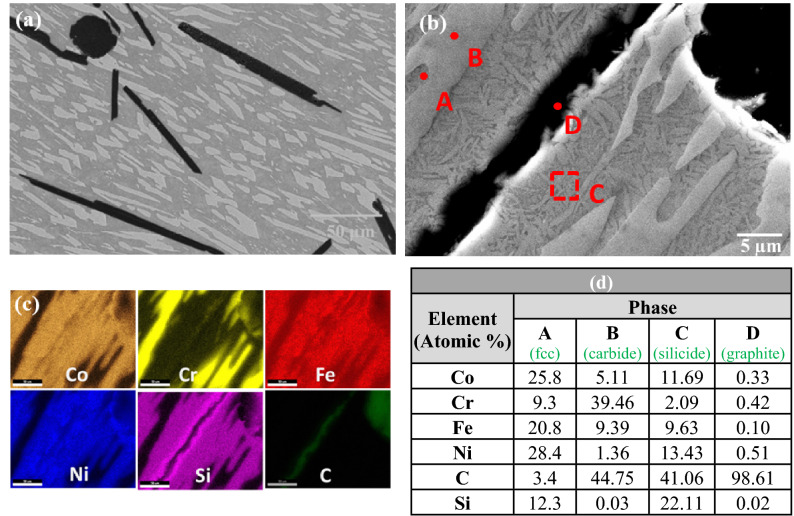
(**a**) SEM image of sample S-5, (**b**) magnified view, (**c**) elemental mapping, and (**d**) EDS results at the indicated positions.

**Figure 7:**
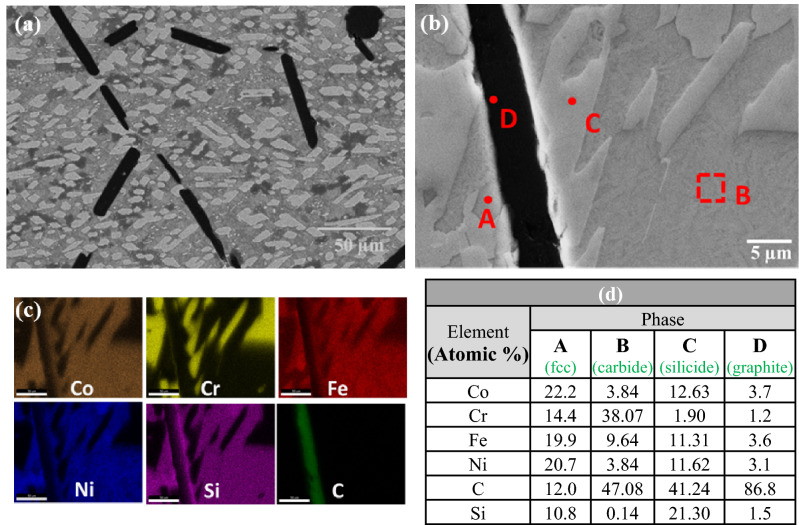
(**a**) SEM image of sample S-6, (**b**) magnified view, (**c**) elemental mapping, and (**d**) EDS results at the indicated positions.

SEM and EDS mapping results of the S-5 and S-6 composites are shown in Figs. 6 and 7, respectively. The presence of graphite flakes in addition to a small number of globules was also observed. The number of graphite flakes, and the volume fraction of the silicon rich phase, were both greater in the S-6 composite than in the S-5 composite.

**Figure 8:**
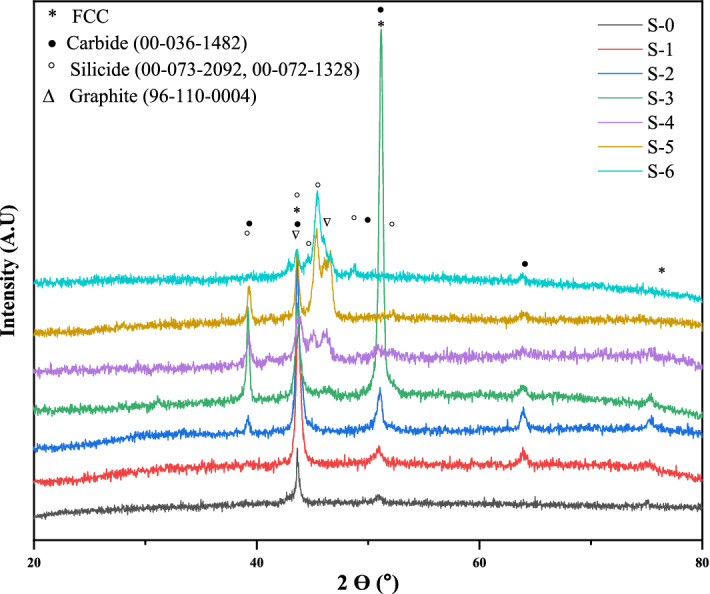
XRD patterns of the as-developed high entropy alloy composites.

Crystal structure characterization of the HEA composites was also carried out using XRD measurements. The results are shown in Fig. 8. Diffraction pattern of the base HEA (S-0) showed the presence only of peaks corresponding to the fcc phase. The presence of additional peaks corresponding to chromium carbide (Cr_7_C_3_) were found in the XRD patterns of the S-1, S-2 and S-3 composites, and less strongly in samples S-3 and S-4, consistent also with the EDS data seen for these samples. Peaks corresponding to Co/Ni silicides were observed in the case of S-3 and S-4 samples, again in agreement with the EDS mapping results shown in Figs. 3 and 4. Peaks corresponding to graphite were observed in the XRD patterns of samples S-5 and S-6.

The microstructure and crystal structure characterization of the as-developed composites both indicate decomposition of the added SiC. This is attributed to the presence of chromium in the HEA matrix. Chromium has a very strong affinity towards carbon^54,55^ and interacts with free carbon to form carbides, as indicted by the observed decrease in the amount of chromium in the matrix. As a result of the dissociation of SiC, the Si goes into the fcc phase^56^. Increasing the amount of SiC addition to the base HEA results therefore in an increase in the amount of carbide phases and the amount of free Si in the microstructure. This additional Si is found to be accommodated in the matrix at low concentrations (in the S-1 and S-2 composites), whereas at higher concentrations (the S-3 to S-6 composites) this leads to additional precipitation of cobalt/nickel silicides. The standard enthalpies of formation of Co and Ni silicides, obtained by high temperature direct synthesis calorimetry, are − 37.9 ± 2.0, − 49.3 ± 1.3, − 34.9 ± 1.1 kJ mol ^−1^ for Co_2_Si, CoSi and CoSi_2,_ respectively, while the values for Ni_2_Si and Ni_5_Si_2_ are − 50.6 ± 1.7 and − 45.1 ± 1.4 kJ mol^−1^, respectively^57^. These values are lower than the heat of formation for SiC, indicating that dissociation of SiC leading to the formation of cobalt/nickel silicide is energetically favorable. In the S-5 composites and S-6 composites, additional free silicon in excess of that taken up by the formation of silicide is present. This free Si was found to promote graphitization, as observed also in conventional steels^58^.

**Figure 9:**
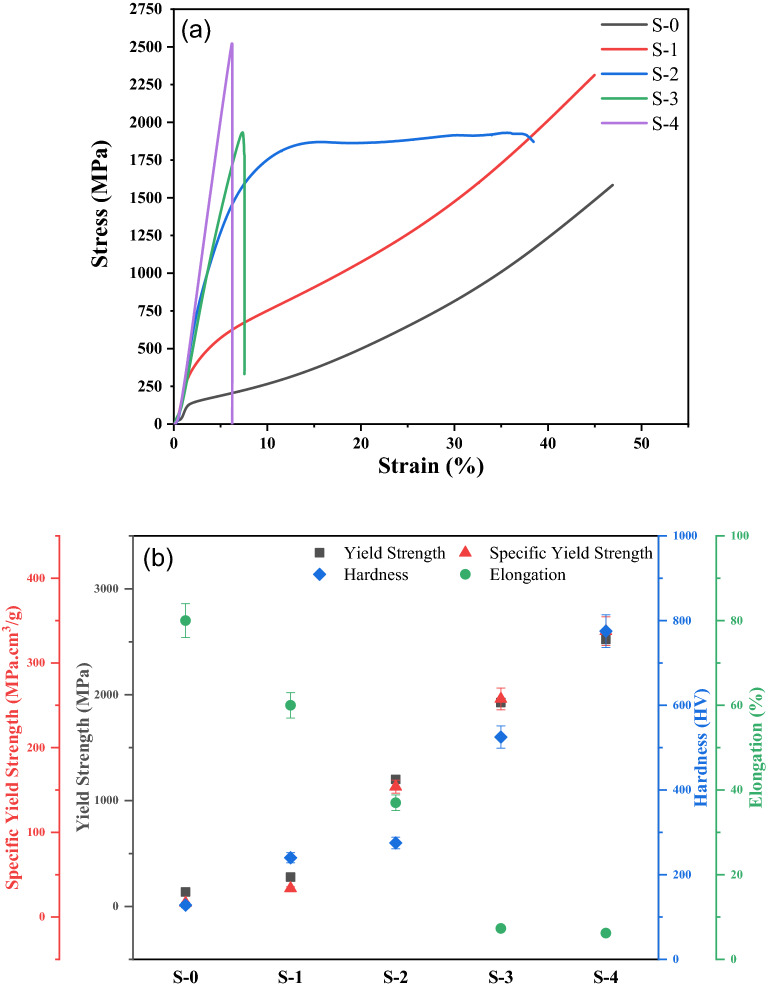
(**a**) Compressive stress–strain curves and (**b**) scatter plot showing specific yield strength, yield strength, hardness and elongation. Note that data for only samples S-0 to S-4 are shown, as samples S-5 and S-6 contained obvious casting defects.

The mechanical behavior of the as-developed ceramic-reinforced HEA matrix composites was studied by performing compression tests and hardness testing. Stress–strain curves of the as-developed composites are shown in Fig. 9a, while Fig. 9b showing scatter plot between specific yield strength, yield strength, hardness and elongation of developed composite.

**Figure 10:**
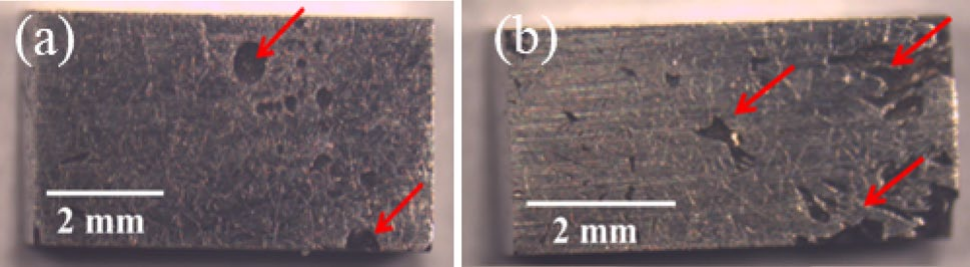
Stereo micrographs showing casting defects (indicated by red arrows) in the S-5 and S-6 composite samples.

It is seen in Fig. 9 that the yield strength increases from 136 MPa for the base HEA (S-0) to 2522 MPa for the S-4 composite. The S-2 composite exhibits a very good elongation before fracture of ~ 37%, while also showing a significantly higher value of yield strength (1200 MPa) in comparison to the base HEA. The excellent combination of strength and ductility in this composite is attributed to the overall microstructural refinement, including a uniform distribution of fine carbide platelets throughout the microstructure, which are expected to hinder the movement of dislocations. The yield strengths of the S-3 and S-4 composites are found to be 1925 MPa and 2522 MPa, respectively. These high values of yield stress can be attributed to a high-volume fraction of hard carbide and silicide phases. The presence of these phases also, however, contributes to a low elongation before fracture of just 7%. The stress strain curve of the base CoCrFeNi HEA (S-0) and S-1 composites show a convex appearance, which is a characteristic feature indicating activation of either twinning or TRIP effects^59,60^. In contrast to sample S-1, the stress–strain curve of sample S-2 exhibits a concave shape up to a strain of approx. 20%, i.e., indicating conventional dislocation glide as the primary deformation mode in this sample in that strain regime^60,61^. The work-hardening rate in this sample nevertheless remains high over a large strain range, and at higher strains a transition to a convex appearance is also seen (though it cannot be ruled out that this is related to a break-down of lubrication conditions during compression loading). The S-3 and S-4 composites show only limited plasticity due to the presence of higher volume fractions of carbides and silicides in the microstructure. Compression testing of the S-5 and S-6 composite samples was not carried out due to the presence of obvious casting defects in these composite samples (see Fig. 10).

The hardness measurements of the HEA composites are shown in Fig. 9b. The hardness of the base HEA was found to be 130 ± 5 HV, while the hardness values of samples S-1, S-2, S-3 and S-4 were found to be 250 ± 10 HV, 275 ± 10 HV, 570 ± 20 HV and 755 ± 20 HV, respectively. The increase in hardness tracks reasonably well the variation in yield stress obtained from compression testing and is attributed to the formation of an increasing amount of hard phases in the composites. The specific yield strength, calculated based on the target composition of each sample, is also shown in Fig. 9b. Overall, the best combination of yield strength (1200 MPa), hardness (275 ± 10 HV) and elongation before fracture (~ 37%) was observed for the S-2 composite.

**Figure 11:**
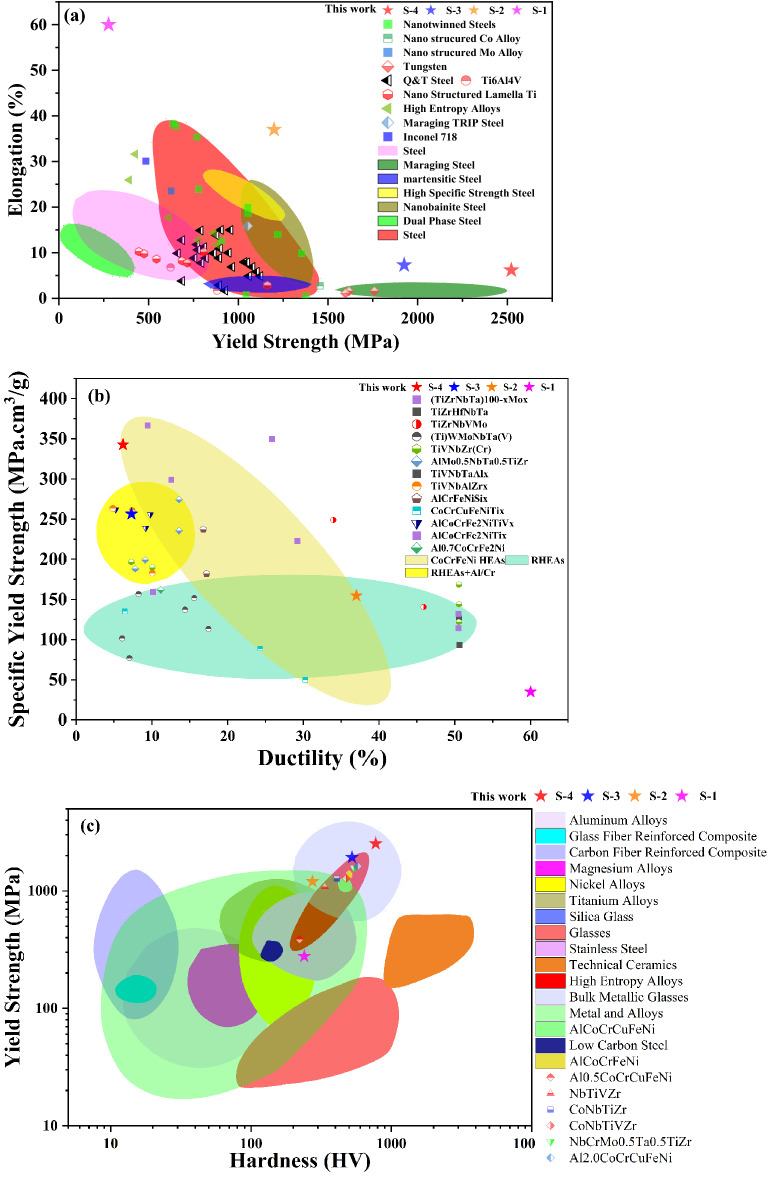
Comparison of results of the present study with different engineering materials and HEAs: (**a**) Elongation versus yield strength^62^, (**b**) specific yield strength versus ductility^63^, and (**c**) yield strength versus hardness^65^.

A comparison of the yield strength and elongation of the as-developed composites with different classes of materials is shown in Fig. 11a. The CoCrFeNi-based composites in the present study show higher values of elongation at any given stress level^62^. It can be seen also that the properties of the HEA composites developed in the present study lie in previously unoccupied regions of the yield strength versus elongation map. Moreover, the as-developed composites exhibit a wide range of strength (277 MPa, 1200 MPa, 1925 MPa and 2522 MPa) and elongation (> 60%, 37%, 7.3% and 6.19%) combinations. Specific yield strength is also an important consideration in shortlisting materials for advanced engineering applications^63,64^. In this regard, the present HEA composites exhibit excellent combinations of specific yield strength and elongation. This is because addition of low density SiC results in composites with high specific yield strength. The specific yield strength and elongation of the HEA composites lie in the same range as that for FCC HEAs as well as for refractory HEAs, as shown in Fig. 11b. The hardness and yield strength of the developed composites lie in the same range as found for bulk metallic glasses^65^ (Fig. 11c). High hardness and yield strength are characteristic features of bulk metallic glasses (BMGs), but they exhibit limited elongation^66,67^. The hardness and yield strength of some of the HEA composites developed in the present study, however, also exhibit significant elongation. It is therefore concluded that the as-developed HEA composites offer a unique and a highly sought-after package of mechanical properties that can be useful for different structural applications. This unique combination of mechanical properties can be attributed to the uniform dispersion of hard carbides formed in-situ in the FCC HEA matrix. Microstructural modification resulting from the addition of ceramic phase needs, however, to be carefully studied and controlled to avoid casting defects, such as those found for the S-5 and S-6 composites, as part of the goal to obtain better combinations of strength and ductility.

## Conclusions

The microstructure and mechanical properties of a series of HEA-ceramic composites, based on the CoCrFeNi HEA system with increasing additions of SiC, have been investigated, yielding the following conclusions:

1. High entropy alloy composites can be successfully developed by addition of SiC to the CoCrFeNi HEA using an arc melting route.
2. SiC decomposes during arc melting resulting in the in-situ development of carbides, silicides, and graphite phases, with the presence and volume fraction of these phases depending on the amount of SiC added to the base HEA.
3. The HEA composites exhibit a wide range of outstanding mechanical properties and possess properties falling in a previously unoccupied region in a yield strength versus elongation map. The yield strength of the HEA-composite prepared using 6 wt.% SiC is more than eight times larger than that of the base HEA, while still retaining 37% ductility.
4. The hardness and yield strength of the HEA-composites lie in the range of bulk metallic glasses (BMGs).

The results of the investigation demonstrate that high entropy alloy composites represent a promising approach towards the goal of achieving outstanding combinations of mechanical properties in metals for advanced structural applications.

## Data Availability

The datasets used and/or analyzed during the current study are available from the corresponding author on reasonable request.
